# AGE-RELATED DIFFERENCES IN HEALTH-RELATED QUALITY OF LIFE AMONG WESTERN CANADIAN NURSING HOME RESIDENTS

**DOI:** 10.1093/geroni/igac059.2572

**Published:** 2022-12-20

**Authors:** Bianca Shieu, Todd Schwartz, Matthias Hoben, Mark Toles, Anna Beeber, Ruth Anderson

**Affiliations:** University of Pittsburgh, Pittsburgh, Pennsylvania, United States; UNC Chapel Hill, Chapel Hill, North Carolina, United States; University of Alberta, Edmonton, Alberta, Canada; UNC Chapel Hill, Chapel Hill, North Carolina, United States; Johns Hopkins University, Baltimore, Maryland, United States; UNC Chapel Hill, Chapel Hill, North Carolina, United States

## Abstract

Nursing homes (NHs) typically focus on health-related quality of life (HRQoL) among residents aged 65 and over despite approximately 7% of NH residents are younger (aged 18-64). Research suggests that the needs of younger NH residents are not being met and they may have low HRQoL. However, differences in HRQoL of younger and older NH residents may not be apparent in studies that use HRQoL measures designed for research with older NH residents. We hypothesized that the younger residents would have lower HRQoL mean scores than the older (aged ≥ 65) residents using a HRQoL measure based on the HRQoL score derived from Resident Assessment Instrument – Minimum Data Set 2.0 items. The measure uses items that emphasize physical aspects of quality of life rather than social aspects. In a sample of 21,129 residents from 94 NHs in Western Canada, we performed descriptive analyses, t-test, chi-square test, and an adjusted propensity score (PS) analysis through retrospective cohort study from years 2016 to 2017. The HRQoL index score ranged from -.351 to .996 (Mean= 0.693, SD=0.265). In the PS model, the adjusted mean score for younger was higher than for older adults with a mean difference at 0.061 (95% CI 0.031, 0.091) (p<.001). Other domains such as mental health condition of quality of life must be examined in younger NH residents because it is a crucial factor influencing their daily lives, thereby we can explore a more complete set of HRQoL domains of them and redesign care for their unique needs.

